# Efficacy and safety of HAIC combined with tyrosine kinase inhibitors *versus* HAIC monotherapy for advanced hepatocellular carcinoma: a multicenter propensity score matching analysis

**DOI:** 10.3389/fphar.2024.1410767

**Published:** 2024-07-31

**Authors:** Miaomiao Yang, Xiongying Jiang, Huan Liu, Qingyu Zhang, Jing Li, Li Shao, Lei Zhao

**Affiliations:** ^1^ Department of Oncology, The Affiliated Yantai Yuhuangding Hospital of Qingdao University, Yantai, China; ^2^ Department of Interventional Radiology, Sun Yat-sen Memorial Hospital, Sun Yat-sen University, Guangzhou, China; ^3^ Department of Interventional Radiology, The Third Affiliated Hospital of Sun Yat-sen University, Guangzhou, China; ^4^ Department of Integrated Chinese and Western Medicine, The Affiliated Yantai Yuhuangding Hospital of Qingdao University, Yantai, China

**Keywords:** hepatocellular carcinoma, hepatic arterial infusion chemotherapy, tyrosine kinase inhibitors, propensity score matching, combination therapy

## Abstract

**Purpose:**

This study aimed to assess the clinical efficacy and safety of the combined approach involving hepatic arterial infusion chemotherapy (HAIC) and tyrosine kinase inhibitors (TKIs) for the treatment of advanced hepatocellular carcinoma (HCC).

**Patients and methods:**

In this multicenter retrospective study conducted from January 2020 to December 2023, we reviewed advanced HCC patients who were treated either with HAIC alone or with a combination of HAIC and TKIs. To address initial disparities between the two groups, we employed propensity score matching (PSM). Tumor response evaluation was performed following RECIST 1.1 criteria. We compared survival outcomes, including overall survival (OS), progression-free survival (PFS), and objective response rate (ORR), between the two treatment groups. Safety assessments were conducted for all patients.

**Results:**

Following the eligibility review, 138 patients underwent combined treatment with HAIC and TKIs (HT group), while 198 patients received HAIC monotherapy (HA group) and met the inclusion criteria for enrollment in this study. After PSM, 107 patients were assigned to each group. The HT group exhibited a longer median OS (18.0 *versus* 8.8 months; hazard ratio [HR], 0.52, p < 0.001) compared to the HA group. Median PFS was also longer in the HT group, although without statistical significance (6.0 *versus* 4.7 months; HR, 0.85, p = 0.265). The HT group demonstrated a higher ORR (41.1% *versus* 25.2%; p = 0.020). No significant differences were observed between the two groups in the incidence of all adverse events (AEs) or grade 3/4 AEs (any grade: 81.2% for HT *versus* 78.8% for HA, p = 0.68; grade 3/4: 18.1% for HT *versus* 13.6% for HA, p = 0.29). Importantly, all AEs were manageable and acceptable. Notably, no grade 5 AEs occurred in either group.

**Conclusion:**

Combination therapy involving HAIC and TKIs effectively prolonged survival in advanced HCC patients. It represented a preferable alternative to HAIC monotherapy, with manageable safety.

## 1 Introduction

Hepatocellular carcinoma (HCC) ranks as the sixth most common cancer globally and stands as the third leading cause of cancer-related mortality (Sung et al., 2021). Due to its highly asymptomatic nature, over 60% of cases progress to an advanced stage upon diagnosis, precluding curative interventions (Cappuyns et al., 2024). The prognosis for these patients is exceedingly grim, with a median overall survival (OS) of approximately 2.7–4.0 months in the absence of treatment (Lu et al., 2019). Consequently, there is a pressing need for potent therapies that can significantly extend survival.

The Barcelona Clinic Liver Cancer (BCLC) staging system recommends systemic therapy involving targeted therapy and immunotherapy for advanced HCC patients at BCLC stage C (Reig et al., 2022). Based on the promising outcomes from the IMbrave150 trial, guidelines now advocate for atezolizumab-bevacizumab combination therapy as the first-line treatment choice for advanced HCC patients. However, multi-tyrosine kinase inhibitors (TKIs) such as lenvatinib and sorafenib remain viable options if the combination therapy is contraindicated (Trevisani et al., 2024).

In addition to systemic therapies, FOLFOX-based hepatic arterial infusion chemotherapy (HAIC) has demonstrated superior outcomes compared to sorafenib for advanced HCC (Lyu et al., 2022). HAIC delivers a concentrated dose of medication directly to liver tumors, resulting in a significant local antitumor effect, and has been widely used in primary and metastatic hepatic malignant tumors (Sidaway, 2022a). According to Chinese and Japanese guidelines, HAIC is the recommended treatment for advanced HCC, particularly in patients with major portal vein tumor thrombosis (PVTT) (Chen et al., 2020). While a phase III trial has reported survival benefits from combining sorafenib with HAIC using a modified FOLFOX regimen compared to sorafenib monotherapy, the outcomes of HAIC plus TKIs *versus* HAIC monotherapy remain unexplored (He et al., 2019).

In this retrospective study, we aimed to investigate the efficacy of combined HAIC and TKIs compared to HAIC monotherapy in advanced HCC patients.

## 2 Material and methods

### 2.1 Study design and patients

In this retrospective study, we investigated advanced HCC patients who received HAIC either combined with or without TKIs between January 2020 and December 2023 at three teaching hospitals in China. The patients were divided into two groups: the HA group (HAIC monotherapy) and the HT group (HAIC combined with TKIs).

The inclusion criteria were as follows:

1. Aged 18–75 years
2. Radiologically or pathologically diagnosed HCC according to the American Association for the Study of Liver Diseases (AASLD) practice guidelines
3. Classified as BCLC C stage
4. Without prior treatment for HCC
5. Child-Pugh A or B, and Eastern Cooperative Oncology Group Performance Status score (ECOG PS) of 0–1
6. No other malignancies within the past 5 years
7. Received at least two cycles of HAIC
8. TKIs included only sorafenib and lenvatinib
9. Complete medical and follow-up data available

The exclusion criteria included:

1. Insufficient organ function or inadequate hematologic function
2. Severe underlying cardiac, pulmonary, or renal disease
3. Discontinue treatment without progression or unacceptable toxicities
4. Loss to follow up

Laboratory tests and imaging evaluations, including enhanced computed tomography (CT) or magnetic resonance imaging (MRI), were performed within a week before the initial treatment. The study received approval from the Ethics Committee of the three teaching hospitals, and informed consent was waived due to the retrospective nature of the study.

### 2.2 Treatment procedures

#### 2.2.1 HAIC treatment

HAIC was administered following previously described protocols (Lyu et al., 2022). Briefly, based on tumor size, location, and arterial supply, the catheter tip was meticulously inserted into branches of the tumor-feeding hepatic artery. The HAIC regimen included oxaliplatin (130 mg/m^2^, administered from hour 0 to 2 on day 1), leucovorin (200 mg/m^2^, administered from hour 2 to 4 on day 1), and fluorouracil (400 mg/m^2^ fluorouracil bolus within 15 min, followed by 2,400 mg/m^2^ fluorouracil maintenance over 46 h on days 1 and 2). After completion of chemotherapy, the catheter and sheath were removed. Repeated HAIC sessions were scheduled at 3-week intervals based on operator evaluation, with no more than 8 cycles.

#### 2.2.2 Lenvatinib treatment

Lenvatinib was administered once daily (12 mg to patients over 60 kg and 8 mg to patients under 60 kg) 3–5 days after the first HAIC session. If grade 1/2 AEs occurred, the dosage was not adjusted, and symptomatic treatment was provided to deal with the AEs as soon as possible. If grade 3/4 AEs took place, the dose was reduced to 8 mg and 4 mg, respectively, or the frequency was reduced to once every 2 days until the AEs were resolved or alleviated. If the AEs persisted, lenvatinib was suspended until they were alleviated or disappeared.

#### 2.2.3 Sorafenib treatment

Sorafenib was administered 400 mg twice a day, and 3–5 days after the first HAIC session. If grade 1/2 adverse events (AEs) occurred, the dosage was not adjusted, and symptomatic treatment was provided to deal with the AEs as soon as possible. If grade 3/4 AEs took place, the dose was reduced to 200 mg twice a day, or the frequency was reduced to 400 mg once a day until the AEs were resolved or alleviated. If the AEs persisted, sorafenib was suspended until they were alleviated or disappeared.

The opportunity for conversion to salvage liver resection was determined by a multidisciplinary team (MDT) and performed by experienced surgeons with substantial expertise in hepatic resection. Adverse events (AEs) of grade 1 or 2 were promptly managed without altering treatment regimens. In cases of grade ≥3 AEs, the regimen was adjusted until the AEs resolved or were alleviated. If these AEs persisted, treatment was discontinued until resolution.

### 2.3 Assessment and outcomes

Contrast-enhanced CT or MRI scans were conducted every two cycles by two independent experienced radiologists. Any discrepancies in assessment results were resolved through consensus. Treatment response, overall response rates (ORR), and disease control rates (DCR) were evaluated based on the response evaluation criteria in solid tumors (RECIST) version 1.1. Specifically:

• ORR: Defined as the proportion of patients achieving complete response (CR) or partial response (PR).
• DCR: Defined as the proportion of patients achieving CR, PR, or stable disease (SD).
• OS: Measured from admission to death from any cause.
• PFS: Measured from admission to disease progression or death from any cause, whichever occurred first.
• AEs during treatment were recorded and graded according to CTCAE version 5.0.

### 2.4 Propensity score matching analysis

Propensity score matching (PSM) analysis was employed to mitigate selection bias and harmonize patient characteristics. Stepwise logistic regression was utilized to identify variables associated with treatment selection, including age, sex, ECOG-PS, etiology, Albumin-Bilirubin (ALBI) score, Child-Pugh class, tumor size, tumor number, alpha-fetoprotein (AFP) levels, PVTT, and extrahepatic metastasis. A one-to-one nearest-neighbor matching algorithm with a caliper of 0.2 (without replacement) was applied.

### 2.5 Statistical analysis

Statistical analyses were conducted using R statistical software (version 4.0.3; R Foundation Inc., Vienna, Austria) and SPSS version 27.0 (SPSS, Chicago, IL, USA). Continuous variables were expressed as either mean ± standard deviation or median (interquartile range, IQR), and comparisons were made using the Student’s t-test or Mann-Whitney U test. Categorical variables were presented as counts and percentages, and their differences were assessed using the χ^2^ test or Fisher’s exact test. The Kaplan-Meier method was employed for analyzing time-to-event variables, and differences were evaluated using the log-rank test. Univariable and multivariable Cox regression analyses were performed to identify factors associated with survival. Variables with a P-value <0.1 in univariate analysis were included in the multivariate analysis. A two-sided P-value <0.05 was considered statistically significant.

## 3 Results

### 3.1 Patient characteristics

**FIGURE 1:**
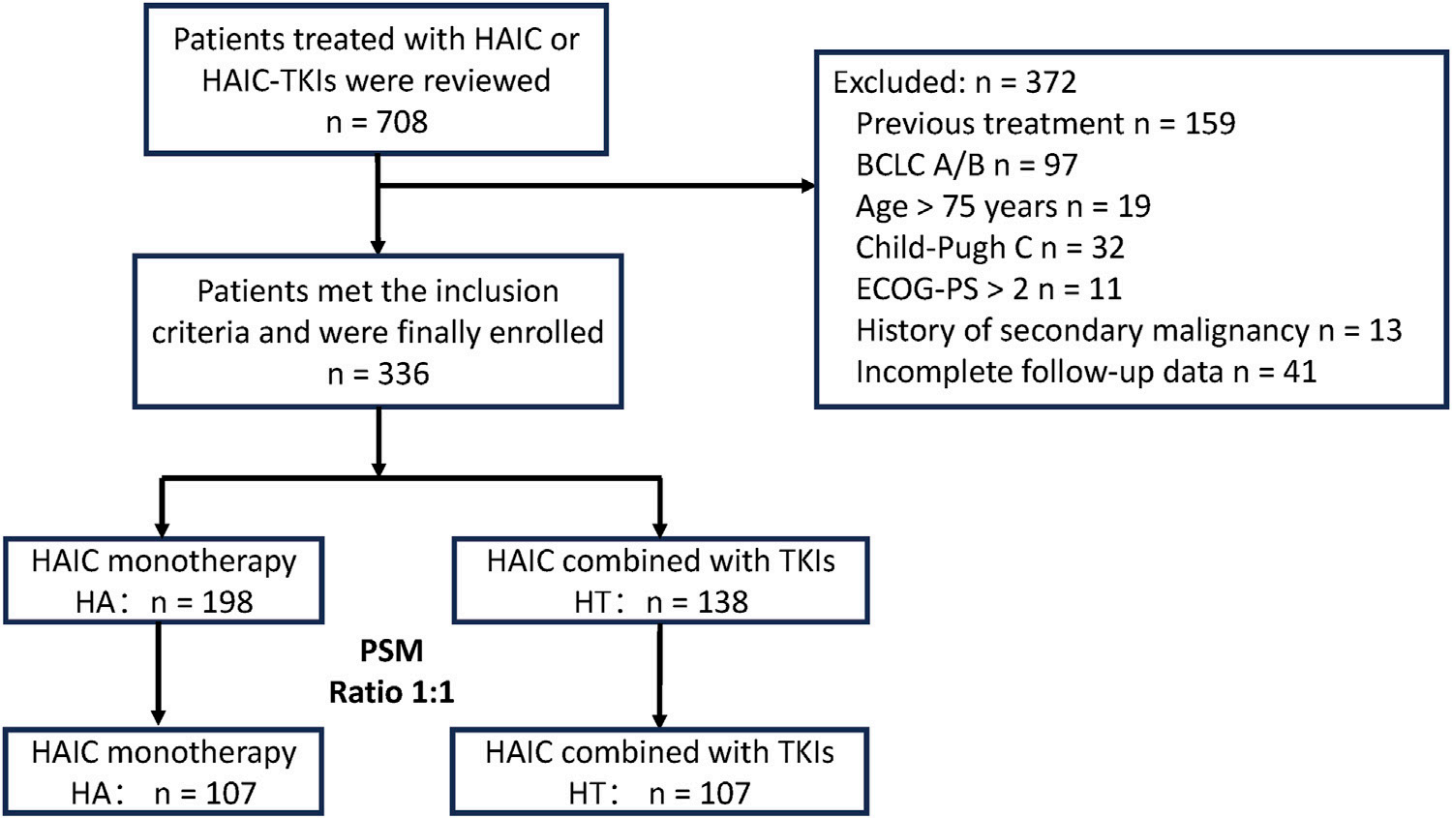
Patient flowchart. PSM, propensity score matching; HT, HAIC combined with TKIs; HAIC: hepatic arterial infusion chemotherapy; TKIs, tyrosine kinase inhibitors; BCLC, Barcelona Clinic Liver Cancer; ECOG, Eastern Cooperative Oncology Group; PS, performance score.

The flowchart depicting patient selection is illustrated in Figure 1. A total of 336 patients with advanced HCC underwent eligibility assessment. Among them, 198 patients received HAIC monotherapy (HA group), while 138 patients received combination therapy (HT group), with median follow-up periods of 65.8 and 41.6 months, respectively. Sex, ECOG-PS, and tumor number significantly differed between the two groups. Consequently, PSM generated 107 patients in each group to mitigate selection bias. Baseline patient characteristics were well-matched between the groups after PSM (all p > 0.2) (Table 1). Besides, the number of patients and the baseline characteristics from different centers were compared in Supplementary Table S1.

**TABLE 1:** Baseline characteristics of the study patients before and after PSM.

Characteristics	Before matching			After matching		
	HA (n = 198)	HTs (n = 138)	P	HA (n = 107)	HT (n = 107)	P
Age (mean ± SD, year)	50 ± 11	52 ± 12	0.058	50 ± 11	51 ± 12	0.357
<60y	157 (79%)	99 (72%)	0.110	86 (80%)	81 (76%)	0.409
≥60y	41 (21%)	39 (28%)		21 (20%)	26 (24%)	
Sex			0.028			0.603
Male	174 (88%)	131 (95%)		98 (92%)	100 (93%)	
Female	24 (12%)	7 (5%)		9 (8%)	7 (7%)	
ECOG-PS			<0.001			0.665
0	185 (93%)	99 (72%)		94 (88%)	96 (90%)	
1	13 (7%)	39 (28%)		13 (12%)	11 (10%)	
Etiology			0.478			>0.999
HBV	187 (94%)	127 (92%)		100 (93%)	101 (94%)	
HCV	0 (0)	1 (1%)		0 (0)	0 (0)	
No-hepatitis	11 (6%)	10 (7%)		7 (7%)	6 (6%)	
ALBI			0.148			>0.999
1	77 (39%)	46 (33%)		40 (37%)	41 (38%)	
2	121 (61%)	90 (65%)		67 (63%)	66 (62%)	
3	0 (0)	2 (1%)		0 (0)	0 (0)	
Child-Pugh			0.280			>0.999
A	182 (92%)	122 (88%)		100 (93%)	100 (93%)	
B	16 (8%)	16 (12%)		7 (7%)	7 (7%)	
Size (Mean ± SD, cm)	12.6 ± 9.2	11.9 ± 3.2	0.291	12.0 ± 3.07	12.0 ± 3.25	0.978
<10 cm	60 (30%)	39 (28%)	0.496	27 (25%)	28 (26%)	0.961
10–15 cm	100 (51%)	78 (57%)		63 (59%)	61 (57%)	
≥15 cm	38 (19%)	21 (15%)		17 (16%)	18 (17%)	
Number			<0.001			0.669
Single	44 (22%)	58 (42%)		37 (35%)	40 (37%)	
Multiple	154 (78%)	80 (58%)		70 (65%)	67 (63%)	
AFP (μg/L)			0.453			0.771
≤400	68 (34%)	42 (30%)		36 (34%)	34 (32%)	
>400	130 (66%)	96 (70%)		71 (66%)	73 (68%)	
PVTT			0.391			0.609
Presence	153 (77%)	112 (81%)		84 (79%)	87 (81%)	
Absence	45 (23%)	26 (19%)		23 (21%)	20 (19%)	
Extrahepatic metastasis			0.639			0.891
Presence	117 (59%)	78 (57%)		59 (55%)	58 (54%)	
Absence	81 (41%)	60 (43%)		48 (45%)	49 (46%)	

Values are presented as n (%).

P values were calculated using a two-sided χ^2^ test.

PSM, propensity score matching; HA, HAIC, monotherapy; HAIC, hepatic arterial infusion chemotherapy; HT, HAIC, combined with TKIs; TKIs, tyrosine kinase inhibitors; ECOG, eastern cooperative oncology group; PS, performance score; HBV, hepatitis B virus; HCV, hepatitis C virus; ALBI, albumin-bilirubin; AFP, alpha-fetoprotein; PVTT, portal vein tumor thrombus.

The majority of patients had hepatitis B virus (HBV)-related HCC with preserved liver function. The median tumor diameter in both groups exceeded 10 cm, and most patients had more than one intrahepatic lesion before and after PSM, indicating a substantial tumor burden among the enrolled patients. Prior to PSM, the median number of HAIC sessions per patient in the HA group was 4 (range: 2–8), compared to 3 (range: 2–8) sessions in the HT group. Additionally, the TKIs category in the HT group included sorafenib and lenvatinib, with the number of patients in each group detailed in Supplementary Table S2. No statistically significant differences were observed in OS and PFS between patients receiving HAIC plus sorafenib or lenvatinib before and after PSM (p > 0.05) (Supplementary Figure S1).

### 3.2 Tumor response

Table 2 presents the best tumor responses according to RECIST 1.1 criteria. Prior to PSM, patients in the HT group achieved higher rates of CR (5.8% vs. 1.6%, p = 0.032), PR (34.1% vs. 24.2%, p = 0.033), ORR (39.9% vs. 25.8%, p = 0.006), and DCR (84.8% vs. 73.7%, p = 0.016), with a lower rate of PD (15.2% vs. 26.3%, p = 0.006). After PSM, patients in the HT group continued to exhibit higher rates of PR (36.4% vs. 23.4%, p = 0.026), ORR (41.1% vs. 25.2%, p = 0.020), and DCR (88.8% vs. 74.8%, p = 0.012), with a lower rate of PD (11.2% vs. 25.2%, p = 0.012).

**TABLE 2:** Treatment efficacy evaluated by RECIST 1.1 criteria before and after PSM.

	Before matching			After matching		
	HA (n = 198)	HT (n = 138)	P	HA (n = 107)	HT (n = 107)	P
Complete response	3 (1.6%)	8 (5.8%)	0.032	2 (1.8%)	5 (4.7%)	0.22
Partial response	48 (24.2%)	47 (34.1%)	0.033	25 (23.4%)	39 (36.4%)	0.026
Stable disease	95 (48.0%)	62 (44.9%)	0.088	53 (49.6%)	51 (47.7%)	0.78
Progressive disease	52 (26.3%)	21 (15.2%)	0.016	27 (25.2%)	12 (11.2%)	0.012
Overall response	51 (25.8%)	55 (39.9%)	0.006	27 (25.2%)	44 (41.1%)	0.020
Disease control	146 (73.7%)	117 (84.8%)	0.016	80 (74.8%)	95 (88.8%)	0.012

Summary of best response.

Values are presented as n (%).

P values were calculated using a two-sided χ^2^ test.

PSM, propensity score matching; RECIST, response evaluation criteria in solid tumors; HA, HAIC, monotherapy; HAIC, hepatic arterial infusion chemotherapy; HT, HAIC, combined with TKIs; TKIs, tyrosine kinase inhibitors.

Besides, all cases of CR in the HA group (3 patients) and one representative case of CR in the HT group exhibited in Supplementary Figure S2.

**FIGURE 2:**
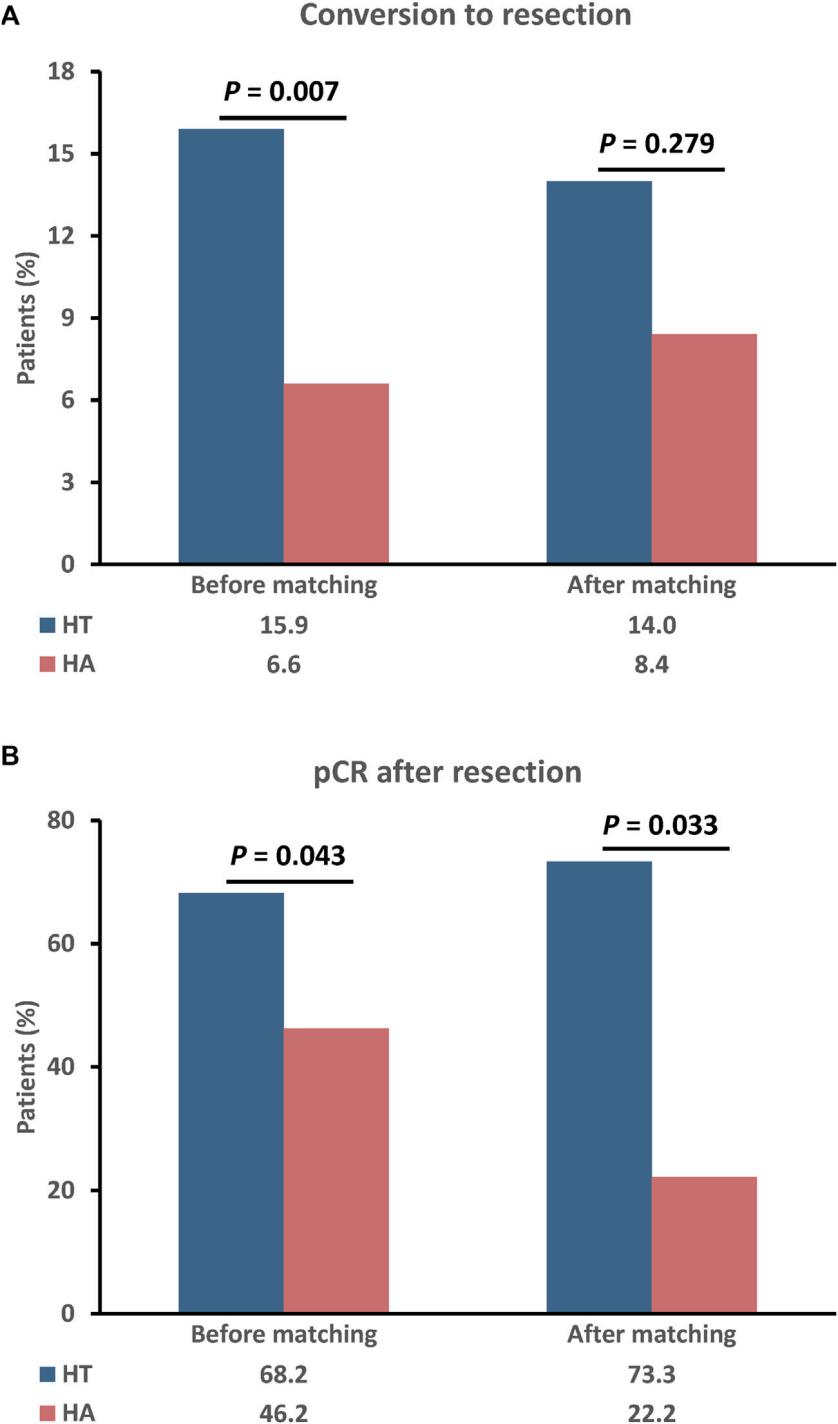
The rate of conversion to resection **(A)** and pCR after resection **(B)** between the two groups before and after PSM. P values were calculated using a two-sided χ^2^ test. PSM, propensity score matching; HT, HAIC combined with TKIs; HAIC: hepatic arterial infusion chemotherapy; TKIs, tyrosine kinase inhibitors.

Figure 2 illustrates patients who successfully underwent surgical resection due to tumor downstaging or significant shrinkage. Prior to PSM, a greater proportion of patients in the HT group received surgical resection compared to the HA group (22/138, 15.9% vs. 13/198, 6.6%; p = 0.007), with a higher rate of pathological complete response (pCR) (15/22, 68.2% vs. 4/13, 46.2%; p = 0.043). After PSM, more patients in the HT group underwent surgical resection (15/107, 14.0% vs. 9/107, 8.4%; p = 0.279), with a higher rate of pCR (11/15, 73.3% vs. 2/9, 22.2%; p = 0.033).

### 3.3 Survival outcomes

**FIGURE 3:**
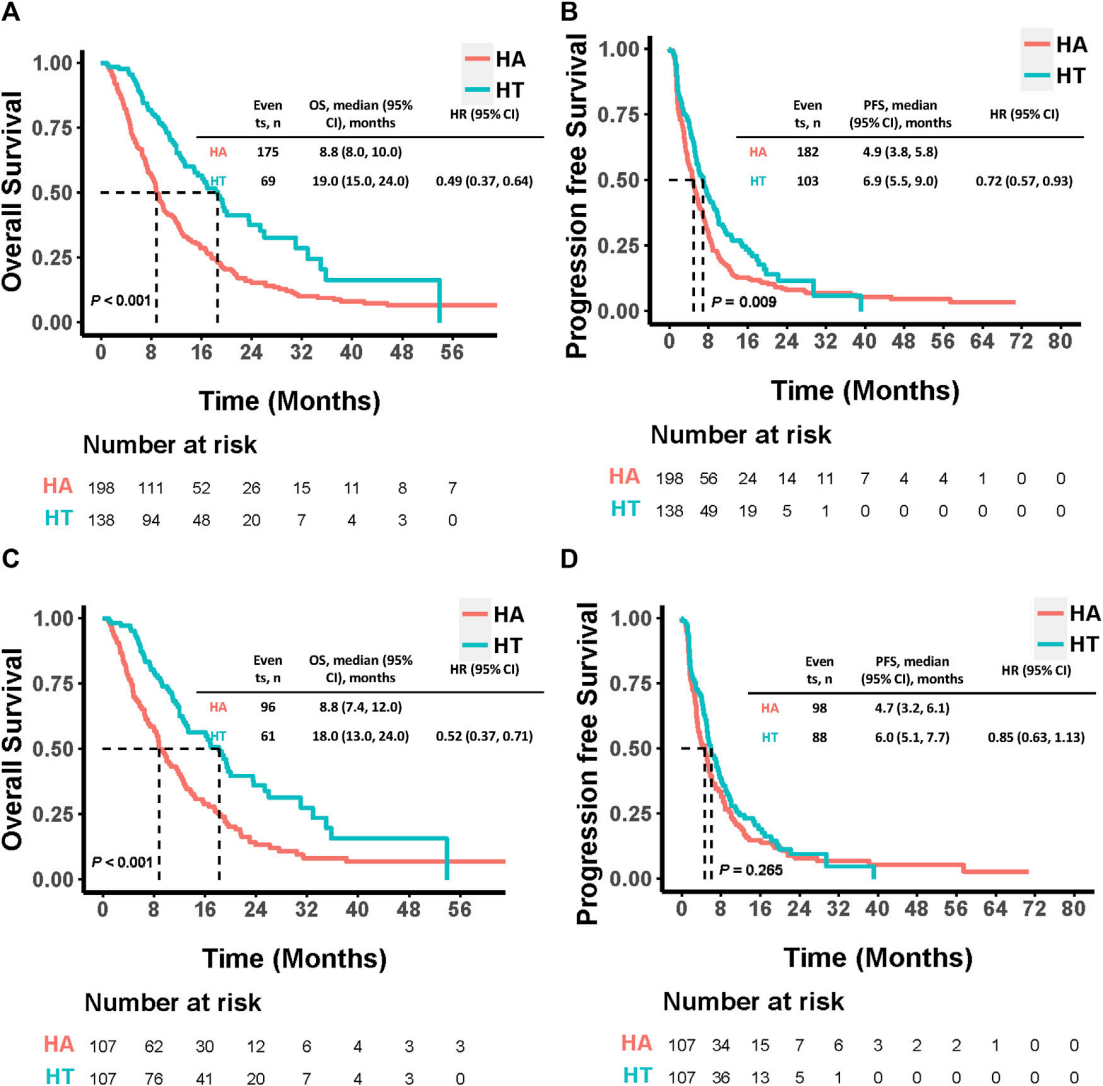
Kaplan-Meier curves comparing OS and PFS among patients who underwent HAIC combined with TKIs *versus* HAIC monotherapy before **(A, B)** and after **(C, D)** PSM. P values were calculated using Log-rank test. PSM, propensity score matching; HT, HAIC combined with TKIs; HAIC: hepatic arterial infusion chemotherapy; TKIs, tyrosine kinase inhibitors; OS, overall survival PFS: progression-free survival; HR: hazard ratio; CI: confidence interval.

Before PSM, 69 patients in the HT group and 175 in the HA group had died by the end of the follow-up period. The median OS was significantly longer in the HT group (19.0 months, 95% CI: 15.0–24.0) compared to the HA group (8.8 months, 95% CI: 8.0–10.0; HR: 0.49, 95% CI: 0.37–0.64, p < 0.001). Additionally, the HT group exhibited a significantly longer median PFS than the HA group (6.9 months, 95% CI: 5.5–9.0 vs. 4.9 months, 95% CI: 3.8–5.8; HR: 0.72, 95% CI: 0.57–0.93, p = 0.009). Among the 107 PSM-matched pairs, the HT group continued to demonstrate significantly longer median OS compared to the HA group (18.0 months, 95% CI: 13.0–24.0 vs. 8.8 months, 95% CI: 7.4–12.0; HR: 0.52, 95% CI: 0.37–0.71, p < 0.001). Although the median PFS was longer in the HT group compared to the HA group, this difference was not statistically significant (6.0 months, 95% CI: 5.1–7.7 vs. 4.7 months, 95% CI: 3.2–6.1; HR: 0.85, 95% CI: 0.63–1.13, p = 0.265) (Figure 3).

In addition, there were total 38 patients who experienced reduction or discontinuation during the treatment period, and 24 patients reduced and 14 patients discontinued in the HT group, respectively. The median overall survival (OS) and median progression-free disease (PFS) were both significant longer in the patients adherence to the TKI in the HT group (OS: 19.3 months, 95% CI: 16.1–35.0 vs. 12.0 months, 95% CI: 9.0–23.5; HR: 0.54, 95% CI: 0.31–0.95, p = 0.013; PFS: 7.6 months, 95% CI: 6.1–10.0 vs. 4.5 months, 95% CI: 2.8–9.2; HR: 0.63, 95% CI: 0.41–0.95, p = 0.029) (Supplementary Figure S3).

### 3.4 Univariate and multivariate analysis

Univariate and multivariate analyses were performed to identify predictors of OS and PFS, and the results are presented in Table 3. Multivariate Cox regression analysis revealed that combination therapy was an independent risk factor for both OS (HR: 0.49; 95% CI: 0.36–0.65, p < 0.001) and PFS (HR: 0.76; 95% CI: 0.59–0.98, p < 0.032). Additionally, ALBI score, AFP level, Vp3/4 PVTT and metastasis were independent risk factors for OS, while metastasis remained an independent risk factor for PFS.

**TABLE 3:** Univariate and multivariate analyses of predictors of survival after treatment.

Characteristics	Overall survival				Progression-free survival			
	Univariate analysis		Multivariate analysis		Univariate analysis		Multivariate analysis	
	HR (95% CI)	P	HR (95% CI)	P	HR (95% CI)	P	HR (95% CI)	P
Treatment (HT)	0.49 (0.37–0.64)	<0.001	0.49 (0. 37–0.68)	<0.001	0.73 (0.57–0.93)	0.010	0.76 (0.59–0.98)	0.032
Age (≥60y)	0.88 (0.64–1.21)	0.432			0.82 (0.62–1.10)	0.188		
Sex (Female)	0.82 (0.54–1.24)	0.35			0.92 (0.62–1.36)	0.676		
ECOG-PS (1)	0.75 (0.49–1.15)	0.19			0.70 (0.49–1.01)	0.056	0.77 (0.52–1.12)	0.171
Etiology (No-hepatitis)	0.63 (0.35–1.12)	0.12			0.73 (0.45–1.19)	0.202		
ALBI (1)	0.75 (0.58–0.97)	0.031	0.71 (0.55–0.91)	0.009	0.88 (0.69–1.12)	0.299		
Child-Pugh (B)	1.09 (0.68–1.74)	0.726			1.12 (0.75–1.68)	0.576		
Size (cm)								
<10	0.93 (0.70–1.24)	0.626			0.89 (0.68–1.16)	0.375		
≥15	1.28 (0.91–1.81)	0.157			1.16 (0.84–1.60)	0.366		
Number (Single)	0.66 (0.49–0.88)	0.005	0.88 (0.59–1.08)	0.145	0.84 (0.65–1.09)	0.195		
AFP (<400 μg/L)	0.74 (0.57–0.98)	0.032	0.69 (0.51–0.89)	0.004	0.81 (0.63–1.04)	0.097	0.81 (0.63–1.04)	0.100
PVTT	1.00 (0.74–1.35)	0.996			1.02 (0.77–1.36)	0.891		
Vp1/2	1.00 (0.68–1.38)	0.871			1.01 (0.65–1.27)	0.732		
Vp3/4	1.33 (1.09–1.75)	0.035	1.28 (1.08–1.53)	0.048	1.18 (0.91–1.35)	0.189		
Metastasis (Absence)	0.76 (0.59–0.98)	0.033	0.74 (0.57–0.96)	0.023	0.75 (0.59–0.94)	0.015	0.72 (0.57–0.91)	0.006

Univariable and multivariable Cox regression analyses were performed to identify the factors associated with survival. Factors with p < 0.1 in univariate analysis were included in multivariate analysis. Two-sided P< 0.05 was defined as statistically significant.

HA, HAIC, monotherapy; HAIC, hepatic arterial infusion chemotherapy; HT, HAIC, combined with TKIs; TKIs, tyrosine kinase inhibitors; ECOG, eastern cooperative oncology group; PS, performance score; HBV, hepatitis B virus; HCV, hepatitis C virus; ALBI, albumin-bilirubin; AFP, alpha-fetoprotein; PVTT, portal vein tumor thrombus.

### 3.5 Subgroup analysis

**FIGURE 4:**
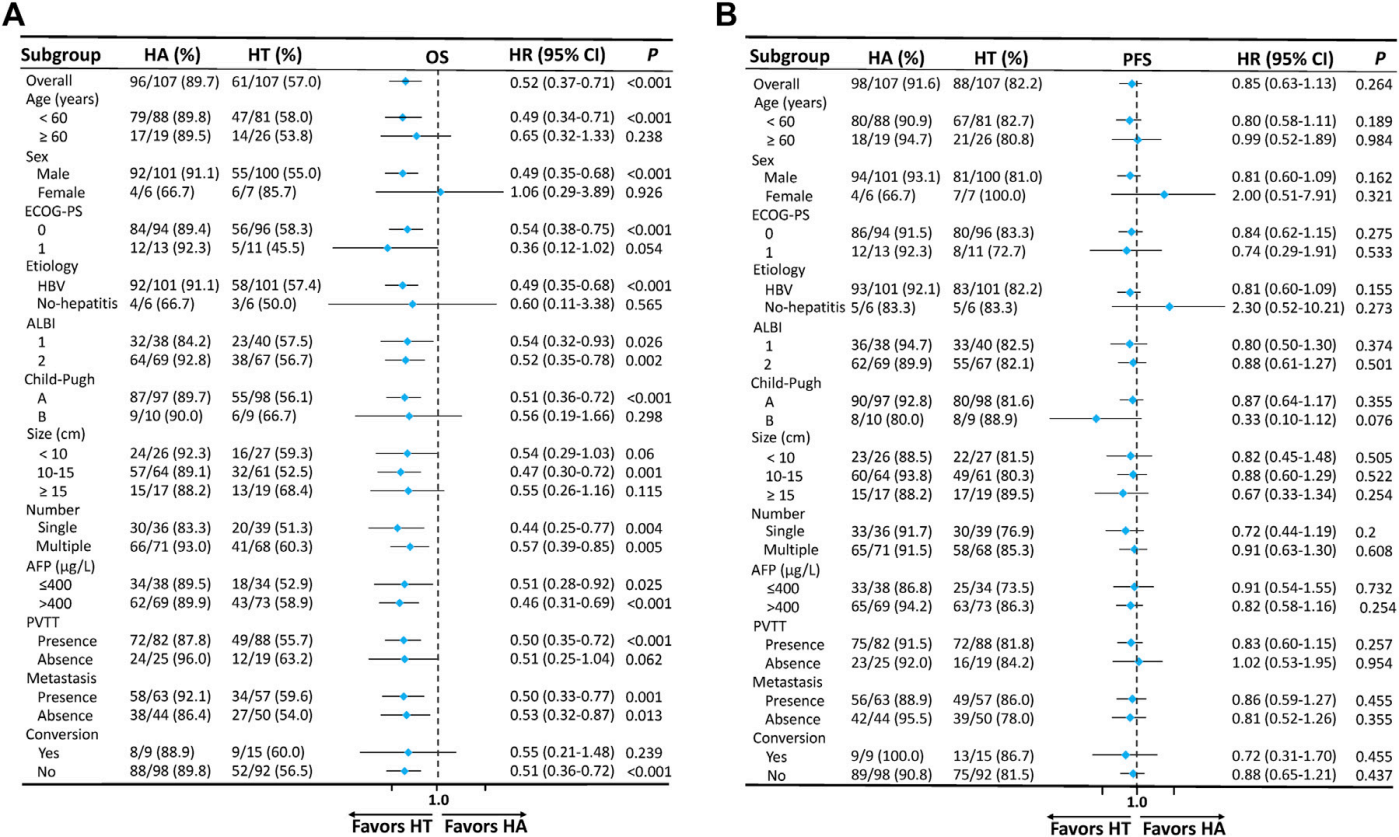
Forest plots based on OS **(A)** and PFS **(B)** of each subgroup. P values were calculated using Log-rank test. PSM, propensity score matching; HT, HAIC combined with TKIs; HAIC: hepatic arterial infusion chemotherapy; TKIs, tyrosine kinase inhibitors; ECOG, Eastern Cooperative Oncology Group; PS, performance score; HBV, hepatitis B virus; ALBI, albumin-bilirubin; AFP, alpha-fetoprotein; PVTT, portal vein tumor thrombus; OS, overall survival PFS: progression-free survival; HR: hazard ratio; CI: confidence interval.

Forest plots (Figure 4) were generated to illustrate the comparison between subgroups. Regarding OS and PFS, it was observed that the HT group consistently demonstrated enhanced benefits across almost all subgroups compared to the HA group. These findings suggested that HAIC combined with TKIs was effective for all analyzed subgroups of advanced HCC patients.

### 3.6 Safety

Treatment-related AEs (TRAEs) are listed in Table 4. The most common TRAEs included nausea, hypoproteinemia, and diarrhea, with most falling into grade 1 or 2. No significant differences were observed in the overall incidence of any grade or grade 3/4 TRAEs. However, TKIs-related AEs were notably more frequent in the HT group, including hypertension, hand-foot skin reaction, dysphonia, proteinuria, bleeding (gingiva), and joint pain. Importantly, these AEs were manageable, and no treatment-related deaths occurred during the treatment.

**TABLE 4:** Treatment-related adverse events.

Adverse event	Any grade			Grade 3/4		
	HA (n = 198)	HT (n = 138)	P	HA (n = 198)	HT (n = 138)	P
Overall incidence	156 (78.8)	112 (81.2)	0.68	27 (13.6)	25 (18.1)	0.29
Abdominal pain	46 (23.2)	39 (28.3)	0.31	5 (2.5)	4 (2.9)	0.54
Nausea	98 (49.5)	62 (44.9)	0.44	10 (5.1)	6 (4.3)	0.49
Diarrhea	51 (25.8)	41 (29.7)	0.46	5 (2.5)	3 (2.2)	0.57
Fever	36 (18.2)	25 (18.1)	0.56	0 (0)	0 (0)	-
Decreased appetite	49 (24.7)	40 (29.0)	0.45	5 (2.5)	4 (2.9)	0.55
Rush	13 (6.6)	15 (10.9)	0.17	0 (0)	2 (1.4)	0.17
Fatigue	25 (12.6)	17 (12.3)	0.54	0 (0)	0 (0)	-
Hypoproteinemia	53 (26.8)	43 (31.2)	0.39	2 (1.0)	1 (0.7)	0.63
Elevated bilirubin	19 (9.6)	8 (5.8)	0.23	0 (0)	0 (0)	-
Elevated ALT	30 (15.2)	22 (15.9)	0.88	1 (0.5)	0 (0)	0.59
Elevated AST	36 (18.2)	26 (18.8)	0.89	1 (0.5)	0 (0)	0.59
Hypothyroidism	3 (1.5)	6 (4.3)	0.17	0 (0)	1 (0.7)	0.41
Neurologic toxicity	38 (19.2)	31 (22.5)	0.49	0 (0)	0 (0)	-
Leukopenia	36 (18.2)	24 (17.4)	0.89	5 (2.5)	3 (2.2)	0.57
Thrombocytopenia	25 (12.6)	19 (13.8)	0.87	3 (1.5)	2 (1.4)	0.67
Hypertension	12 (6.1)	39 (28.3)	<0.001	0 (0)	3 (2.2)	0.068
Hand-foot skin reaction	0 (0)	23 (16.7)	<0.001	0 (0)	3 (2.2)	0.068
Dysphonia	0 (0)	26 (18.8)	<0.001	0 (0)	1 (0.7)	0.41
Proteinuria	2 (1.0)	17 (12.3)	<0.001	0 (0)	2 (1.4)	0.17
Bleeding (gingiva)	6 (3.0)	9 (6.5)	<0.001	1 (0.5)	3 (2.2)	0.19
Joint pain	9 (4.5)	18 (13.0)	0.007	0 (0)	2 (1.4)	0.17

Values are presented as n (%).

P values were calculated using a two-sided χ^2^ test.

HA, hepatic arterial infusion chemotherapy; HT, HAIC, combined with TKIs; TKIs, tyrosine kinase inhibitors; ALT, alanine transaminase; AST, aspartate aminotransferase.

## 4 Discussion

This multicenter retrospective study demonstrated an association between HAIC plus TKIs and extended OS by 9.2 months compared with HAIC monotherapy among patients with advanced HCC. These results were consistent across all subgroup analyses. The combination therapy was also associated with a significant improvement in overall tumor response and PFS compared with HAIC monotherapy. Additionally, more patients in the combination therapy group were able to undergo salvage liver resection after tumor regression or necrosis, with 68.2% of patients achieving pCR. Both HAIC combined with sorafenib or HAIC monotherapy demonstrated acceptable safety profiles. These results demonstrate that HAIC combined with TKIs is superior to HAIC monotherapy in the treatment of advanced HCC.

Controversy persists between Western and Eastern approaches to treating advanced HCC patients. While systemic therapies are recommended as the first-line option in the West, locoregional options such as transarterial chemoembolization (TACE), HAIC, and even surgical resection have been adopted in the East, particularly in China (Lu et al., 2019). The primary reason lies in the high tumor burden observed in Chinese patients, a characteristic consistent with our study. These patients require rapid control, which locoregional approaches can provide. HAIC, delivering high doses of chemotherapy directly through the hepatic artery to the tumor, has demonstrated superior outcomes compared to both TACE and sorafenib in China, elevating its status as a more ambitious treatment option (Li et al., 2022; Lyu et al., 2022). Consequently, Chinese guidelines recommend HAIC as a first-line option for advanced HCC patients, especially those with substantial tumor burden (Sun et al., 2022; Yau et al., 2022; Xie et al., 2023; Zhao et al., 2024). Furthermore, previous studies have highlighted the promising antitumor activity of HAIC combined with sorafenib, surpassing sorafenib monotherapy (He et al., 2019; Zheng et al., 2022). Thus, the combination therapy of HAIC with antiangiogenic agents emerges as a novel treatment strategy for patients with advanced HCC.

Apart from sorafenib, lenvatinib has demonstrated comparable tumor response and survival outcomes in patients with advanced HCC (Vogel et al., 2021). Notably, a recent phase III clinical trial (LANUCH) reported that TACE combined with lenvatinib significantly improved survival compared to lenvatinib monotherapy (Peng et al., 2023). This finding suggests a synergistic antitumor effect between locoregional therapy and lenvatinib. However, the efficacy of lenvatinib in combination with HAIC remains uncertain. In our study, more than 60% of patients in the HT group received lenvatinib plus HAIC, and the outcomes, including OS and PFS, were comparable to those of sorafenib plus HAIC, thus validating the synergistic effect of these two treatment regimens. Additionally, it is important to note that the current study population might have worse basic characteristics than that of the IMbrave 150 trial (Finn et al., 2020), as all patients included in our study were classified as BCLC C stage with high tumor burden. Further studies are needed to compare bevacizumab plus atezolizumab and HAIC combined with TKIs as the first-line treatment for advanced HCC.

A previous study demonstrated that FOLFOX-HAIC yields a favorable tumor response in advanced HCC patients with PVTT (Yuan et al., 2023). On the contrary, it may be not an appropriate choice for treating advanced HCC patients with extrahepatic metastases, as its effect in controlling extrahepatic lesions is limited. However, according to some previous studies, patients with extrahepatic metastases demonstrated inferior tumor response and survival outcomes compared to those without distant metastases, but no statistical difference was observed (Lyu et al., 2019; Ueshima et al., 2020). Besides, numerous research studies and case reports have indicated long-term overall survival (OS) benefits following HAIC therapy in advanced HCC patients with extrahepatic metastases (Kogure et al., 2007; Yoshida et al., 2014). The specific mechanisms underlying these favorable outcomes remain unclear. One speculated reason is that after HAIC treatment, certain chemotherapy drugs enter the bloodstream, thereby reaching distant metastatic lesions and exerting anti-tumor effects (Gao et al., 2018). Moreover, it is widely agreed that the primary causes of death in most HCC patients include liver function failure, upper gastrointestinal bleeding, and progression of intrahepatic lesions. Consequently, controlling extrahepatic lesions has minimal impact on patient survival prognosis. Additionally, in cases where patients experience progression of distant extrahepatic lesions, sequential administration of systemic therapeutic drugs can be employed for their control. Therefore, some patients receiving initial HAIC monotherapy were included in this study as the control group. While in the multivariate analysis, it still revealed that extrahepatic metastases were the sole independent prognostic factors for both OS and PFS. As a systemic therapy option, TKIs exert antitumor effects on both intrahepatic and extrahepatic tumors. Furthermore, TKIs are multi-kinase inhibitors with antiproliferative and antiangiogenic activities, countering hypoxia-induced angiogenesis induced by HAIC (Sidaway, 2022b). Consequently, patients with extrahepatic metastases are more likely to benefit from combination therapy.

In this study, we opted for HAIC combined TKIs as a potent approach to rapidly reduce tumor burden. Previous reports have indicated that TACE combined with HAIC may lead to better OS and a higher conversion rate than TACE monotherapy for advanced HCC patients (Li et al., 2021). Although TACE plus HAIC has been reported as superior to TACE monotherapy in retrospective studies (Li et al., 2021; Yuan et al., 2023), whether this combination provides survival benefits compared to HAIC alone remains unclear—especially considering that HAIC has already demonstrated significant OS improvement over TACE in HCC patients with high tumor burden (Li et al., 2022). Additionally, the impact of TACE-induced vascular embolism on HAIC’s ability to deliver chemotherapeutic drugs through tumor-associated arterial branches remains unknown, necessitating further investigation. Furthermore, the incidence of grade 3–4 AEs and overall AEs was higher with TACE than with HAIC (Li et al., 2022), and the addition of TKIs would likely further increase AE incidence. In the absence of evidence supporting the superiority and safety of TACE combined with HAIC, this combination therapy should be cautiously considered for advanced HCC patients.

Except for in combination of HAIC and TKI, several clinical studies are investigating the efficacy of HAIC combined with TKI and ICI in HCC. Preliminary results indicate that this combination shows promise in improving ORR and OS, comparing with monotherapy or dual combination therapy. For instance, the combination of HAIC with apatinib and camrelizumab has demonstrated good efficacy and tolerability in some patients (Zhang TQ. et al., 2023). Combining HAIC with TKI and ICI can produce a synergistic effect, enhancing overall antitumor efficacy. HAIC reduces tumor burden, TKI inhibits tumor growth, and ICI boosts the immune response against cancer cells. This combination targets the tumor through multiple mechanisms, reducing the likelihood of resistance and tumor escape (Llovet et al., 2022). However, given that most of these studies were retrospective or small-sample phase II clinical trials, the efficacy and safety need to be further validated by large-sample prospective clinical studies. Besides, during our study period, most ICIs were not approved for HCC and thus were not covered by medical insurance in China, which resulted in increasing the financial burden on patients, leading them to refuse immunotherapy. Therefore, ICI was not chosen as a first-line treatment option for patients in this study.

In our retrospective study, we employed PSM to mitigate group differences. However, in the matched cohort of this study, although PFS was longer in the HT group compared to the HA group, there was no statistically significant difference between the two groups. One possible explanation is that combination therapy may rapidly reduce tumor burden but has a limited role in preventing tumor progression and metastasis. This suggests that further refinement of combination therapy is necessary. Regarding subgroup analyses, notably, the integration of TKIs with HAIC demonstrated significantly improved OS and PFS outcomes compared to HAIC monotherapy. These benefits were observed regardless of tumor size, tumor number, or the presence of vascular invasion or extrahepatic metastasis. These findings suggest that combining HAIC with TKIs could offer a superior therapeutic approach for advanced HCC, irrespective of tumor burden. However, it is worth noting that within the female and no-hepatitis subgroups, the combination group did not achieve superior survival outcomes, possibly due to the small sample size. Validation in a larger cohort is warranted for further confirmation.

A previous study has reported that HAIC exhibits a higher ORR compared to TACE based on RECIST 1.1 criteria, resulting in a higher conversion rate for unresectable HCC patients (Deng et al., 2023a). Recent research has highlighted that combination therapy can significantly enhance the likelihood of converting to liver resection after reducing tumor burden resulting in a relatively high rate of pCR (Zhu et al., 2021; Zhang W. et al., 2023; Deng et al., 2023b; Kudo et al., 2023; Vitale et al., 2023; Trevisani et al., 2024). Notably, the conversion rate is closely tied to the ORR within a relatively short timeframe (Chen et al., 2024). In our study, the ORR was markedly higher in the HT group, which also exhibited a greater rate of conversion to liver resection and pCR. These findings suggest that combination therapy may facilitate curative treatment for a larger proportion of patients.

In addition to favorable outcomes, combination therapy led to a moderate increase in the incidence of AEs. Notably, there was a higher occurrence of AEs related to TKIs, consistent with findings from previous trials involving sorafenib and lenvatinib (Llovet et al., 2008; Cheng et al., 2009; Kudo et al., 2018). Fortunately, these AEs were generally manageable with appropriate supportive medications and did not exacerbate the disease or necessitate discontinuation of therapy. Interestingly, the incidence of AEs in the HT group was higher than that observed in patients treated with HAIC plus sorafenib in a previous study (He et al., 2019). This discrepancy might be attributed to the fact that our enrolled patients had more advanced disease and worse baseline characteristics. Another notable HAIC-specific AE was abdominal pain, resulting from arteria vasospasm during oxaliplatin infusion. Unfortunately, there are currently no foolproof methods to completely alleviate this specific pain. However, prescribing pain relief and spasm-relieving medications or adjusting the infusion rate of oxaliplatin may help mitigate the discomfort (Lv et al., 2016; Wu et al., 2021). In summary, the combination of HAIC with TKIs demonstrated a safe and tolerable profile.

Our study has several potential limitations that warrant consideration. First, as a retrospective study, inherent selection bias is unavoidable. Therefore, we employed PSM and subgroup analysis to minimize differences between the two groups. Second, it is essential to emphasize that our study was conducted in China, where HCC patients are predominantly influenced by the HBV. Consequently, our findings may have limitations when extrapolated to HCC patients with different etiologies. Finally, given that our study involved multiple centers, variations in the criteria for HAIC may exist among different institutions.

## 5 Conclusion

In conclusion, when compared to HAIC monotherapy, combination therapy involving HAIC combined with TKIs yielded favorable tumor responses and improved long-term survival outcomes for patients with advanced HCC. Importantly, a substantial number of patients achieved curative liver resection following successful conversion. Consequently, combination therapy emerges as a promising treatment strategy for advanced HCC patients.

## Data Availability

The original contributions presented in the study are included in the article/Supplementary Material, further inquiries can be directed to the corresponding authors.

## References

[B1] Cappuyns S. Corbett V. Yarchoan M. Finn R. S. Llovet J. M. (2024). Critical appraisal of guideline recommendations on systemic therapies for advanced hepatocellular carcinoma: a review. JAMA Oncol. 10, 395–404. 10.1001/jamaoncol.2023.2677 37535375 PMC10837331

[B2] Chen L. T. Martinelli E. Cheng A. L. Pentheroudakis G. Qin S. Bhattacharyya G. S. (2020). Pan-Asian adapted ESMO Clinical Practice Guidelines for the management of patients with intermediate and advanced/relapsed hepatocellular carcinoma: a TOS-ESMO initiative endorsed by CSCO, ISMPO, JSMO, KSMO, MOS and SSO. Ann. Oncol. 31, 334–351. 10.1016/j.annonc.2019.12.001 32067677

[B3] Chen Q. F. Chen S. Chen M. Lyu N. Zhao M. (2024). Improving the conversion success rate of hepatocellular carcinoma: focus on the use of combination therapy with a high objective response rate. J. Clin. Transl. Hepatol. 12, 298–304. 10.14218/JCTH.2023.00403 38426191 PMC10899866

[B4] Cheng A. L. Kang Y. K. Chen Z. Tsao C. J. Qin S. Kim J. S. (2009). Efficacy and safety of sorafenib in patients in the Asia-Pacific region with advanced hepatocellular carcinoma: a phase III randomised, double-blind, placebo-controlled trial. Lancet Oncol. 10, 25–34. 10.1016/S1470-2045(08)70285-7 19095497

[B5] Deng M. Cai H. He B. Guan R. Lee C. Guo R. (2023a). Hepatic arterial infusion chemotherapy versus transarterial chemoembolization, potential conversion therapies for single huge hepatocellular carcinoma: a retrospective comparison study. Int. J. Surg. 109, 3303–3311. 10.1097/JS9.0000000000000654 37578432 PMC10651280

[B6] Deng M. Lei Q. Wang J. Lee C. Guan R. Li S. (2023b). Nomograms for predicting the recurrence probability and recurrence-free survival in patients with hepatocellular carcinoma after conversion hepatectomy based on hepatic arterial infusion chemotherapy: a multicenter, retrospective study. Int. J. Surg. 109, 1299–1310. 10.1097/JS9.0000000000000376 37038994 PMC10389618

[B7] Finn R. S. Qin S. Ikeda M. Galle P. R. Ducreux M. Kim T. Y. (2020). Atezolizumab plus bevacizumab in unresectable hepatocellular carcinoma. N. Engl. J. Med. 382, 1894–1905. 10.1056/NEJMoa1915745 32402160

[B8] Gao J. Zhen R. Liao H. Zhuang W. Guo W. (2018). Pharmacokinetics of continuous transarterial infusion of 5-fluorouracil in patients with advanced hepatocellular carcinoma. Oncol. Lett. 15, 7175–7181. 10.3892/ol.2018.8242 29725440 PMC5920382

[B9] He M. Li Q. Zou R. Shen J. Fang W. Tan G. (2019). Sorafenib plus hepatic arterial infusion of oxaliplatin, fluorouracil, and leucovorin vs sorafenib alone for hepatocellular carcinoma with portal vein invasion: a randomized clinical trial. JAMA Oncol. 5, 953–960. 10.1001/jamaoncol.2019.0250 31070690 PMC6512278

[B10] Kogure T. Iwasaki T. Ueno Y. Kanno N. Fukushima K. Yamagiwa Y. (2007). Complete remission of a case of hepatocellular carcinoma with tumor invasion in inferior vena cava and with pulmonary metastasis successfully treated with repeated arterial infusion chemotherapy. Hepatogastroenterology 54, 2113–2116. 18251171

[B11] Kudo M. Aoki T. Ueshima K. Tsuchiya K. Morita M. Chishina H. (2023). Achievement of complete response and drug-free status by atezolizumab plus bevacizumab combined with or without curative conversion in patients with transarterial chemoembolization-unsuitable, intermediate-stage hepatocellular carcinoma: a multicenter proof-of-concept study. Liver Cancer 12, 321–338. 10.1159/000529574 37901197 PMC10603621

[B12] Kudo M. Finn R. S. Qin S. Han K. H. Ikeda K. Piscaglia F. (2018). Lenvatinib versus sorafenib in first-line treatment of patients with unresectable hepatocellular carcinoma: a randomised phase 3 non-inferiority trial. Lancet 391, 1163–1173. 10.1016/S0140-6736(18)30207-1 29433850

[B13] Li B. Qiu J. Zheng Y. Shi Y. Zou R. He W. (2021). Conversion to resectability using transarterial chemoembolization combined with hepatic arterial infusion chemotherapy for initially unresectable hepatocellular carcinoma. Ann. Surg. Open 2, e057. 10.1097/AS9.0000000000000057 37636551 PMC10455427

[B14] Li Q. J. He M. K. Chen H. W. Fang W. Q. Zhou Y. M. Xu L. (2022). Hepatic arterial infusion of oxaliplatin, fluorouracil, and leucovorin versus transarterial chemoembolization for large hepatocellular carcinoma: a randomized phase III trial. J. Clin. Oncol. 40, 150–160. 10.1200/JCO.21.00608 34648352

[B15] Llovet J. M. Castet F. Heikenwalder M. Maini M. K. Mazzaferro V. Pinato D. J. (2022). Immunotherapies for hepatocellular carcinoma. Nat. Rev. Clin. Oncol. 19, 151–172. 10.1038/s41571-021-00573-2 34764464

[B16] Llovet J. M. Ricci S. Mazzaferro V. Hilgard P. Gane E. Blanc J. F. (2008). Sorafenib in advanced hepatocellular carcinoma. N. Engl. J. Med. 359, 378–390. 10.1056/NEJMoa0708857 18650514

[B17] Lu J. Zhang X. P. Zhong B. Y. Lau W. Y. Madoff D. C. Davidson J. C. (2019). Management of patients with hepatocellular carcinoma and portal vein tumour thrombosis: comparing east and west. Lancet Gastroenterol. Hepatol. 4, 721–730. 10.1016/S2468-1253(19)30178-5 31387735

[B18] Lv N. Kong Y. Mu L. Pan T. Xie Q. Zhao M. (2016). Effect of perioperative parecoxib sodium on postoperative pain control for transcatheter arterial chemoembolization for inoperable hepatocellular carcinoma: a prospective randomized trial. Eur. Radiol. 26, 3492–3499. 10.1007/s00330-016-4207-8 26801163

[B19] Lyu N. Kong Y. Pan T. Mu L. Li S. Liu Y. (2019). Hepatic arterial infusion of oxaliplatin, fluorouracil, and leucovorin in hepatocellular cancer with extrahepatic spread. J. Vasc. Interv. Radiol. 30, 349–357. 10.1016/j.jvir.2018.09.004 30819477

[B20] Lyu N. Wang X. Li J. B. Lai J. F. Chen Q. F. Li S. L. (2022). Arterial chemotherapy of oxaliplatin plus fluorouracil versus sorafenib in advanced hepatocellular carcinoma: a biomolecular exploratory, randomized, phase III trial (FOHAIC-1). J. Clin. Oncol. 40, 468–480. 10.1200/JCO.21.01963 34905388

[B21] Peng Z. Fan W. Zhu B. Wang G. Sun J. Xiao C. (2023). Lenvatinib combined with transarterial chemoembolization as first-line treatment for advanced hepatocellular carcinoma: a phase III, randomized clinical trial (LAUNCH). J. Clin. Oncol. 41, 117–127. 10.1200/JCO.22.00392 35921605

[B22] Reig M. Forner A. Rimola J. Ferrer-Fàbrega J. Burrel M. Garcia-Criado Á. (2022). BCLC strategy for prognosis prediction and treatment recommendation: the 2022 update. J. Hepatol. 76, 681–693. 10.1016/j.jhep.2021.11.018 34801630 PMC8866082

[B23] Sidaway P. (2022a). HAIC-FO improves outcomes in HCC. Nat. Rev. Clin. Oncol. 19, 150. 10.1038/s41571-022-00599-0 35013583

[B24] Sidaway P. (2022b). FOLFOX-HAIC active in large HCC. Nat. Rev. Clin. Oncol. 19, 5. 10.1038/s41571-021-00577-y 34711952

[B25] Sun Y. Zhang W. Bi X. Yang Z. Tang Y. Jiang L. (2022). Systemic therapy for hepatocellular carcinoma: Chinese consensus-based interdisciplinary expert statements. Liver Cancer 11, 192–208. 10.1159/000521596 35949289 PMC9218612

[B26] Sung H. Ferlay J. Siegel R. L. Laversanne M. Soerjomataram I. Jemal A. (2021). Global cancer statistics 2020: GLOBOCAN estimates of incidence and mortality worldwide for 36 cancers in 185 countries. CA A Cancer J. Clin. 71, 209–249. 10.3322/caac.21660 33538338

[B27] Trevisani F. Vitale A. Kudo M. Kulik L. Park J. W. Pinato D. J. (2024). Merits and boundaries of the BCLC staging and treatment algorithm: learning from the past to improve the future with a novel proposal. J. Hepatol. 80, 661–669. 10.1016/j.jhep.2024.01.010 38266658

[B28] Ueshima K. Ogasawara S. Ikeda M. Yasui Y. Terashima T. Yamashita T. (2020). Hepatic arterial infusion chemotherapy versus sorafenib in patients with advanced hepatocellular carcinoma. Liver Cancer 9, 583–595. 10.1159/000508724 33083282 PMC7548914

[B29] Vitale A. Cabibbo G. Iavarone M. Viganò L. Pinato D. J. Ponziani F. R. (2023). Personalised management of patients with hepatocellular carcinoma: a multiparametric therapeutic hierarchy concept. Lancet Oncol. 24, e312–e322. 10.1016/S1470-2045(23)00186-9 37414020

[B30] Vogel A. Qin S. Kudo M. Su Y. Hudgens S. Yamashita T. (2021). Lenvatinib versus sorafenib for first-line treatment of unresectable hepatocellular carcinoma: patient-reported outcomes from a randomised, open-label, non-inferiority, phase 3 trial. Lancet Gastroenterol. Hepatol. 6, 649–658. 10.1016/S2468-1253(21)00110-2 34087115

[B31] Wu Z. Guo W. Chen S. Zhuang W. (2021). Determinants of pain in advanced HCC patients recieving hepatic artery infusion chemotherapy. Invest New Drugs 39, 394–399. 10.1007/s10637-020-01009-x 33006020 PMC7960585

[B32] Xie D. Y. Zhu K. Ren Z. G. Zhou J. Fan J. Gao Q. (2023). A review of 2022 Chinese clinical guidelines on the management of hepatocellular carcinoma: updates and insights. Hepatobiliary Surg. Nutr. 12, 216–228. 10.21037/hbsn-22-469 37124695 PMC10129899

[B33] Yau T. Tai D. Chan S. L. Huang Y. H. Choo S. P. Hsu C. (2022). Systemic treatment of advanced unresectable hepatocellular carcinoma after first-line therapy: expert recommendations from Hong Kong, Singapore, and taiwan. Liver Cancer 11, 426–439. 10.1159/000525582 36158587 PMC9485972

[B34] Yoshida T. Kamada K. Miura K. Goto T. Ohshima S. Sato W. (2014). Successful treatment of hepatocellular carcinoma with lung metastasis using hepatic and bronchial artery infusion chemotherapy. Intern Med. 53, 2493–2497. 10.2169/internalmedicine.53.2957 25366009

[B35] Yuan Y. He W. Yang Z. Qiu J. Huang Z. Shi Y. (2023). TACE-HAIC combined with targeted therapy and immunotherapy versus TACE alone for hepatocellular carcinoma with portal vein tumour thrombus: a propensity score matching study. Int. J. Surg. 109, 1222–1230. 10.1097/JS9.0000000000000256 37026861 PMC10389515

[B36] Zhang T. Q. Geng Z. J. Zuo M. X. Li J. B. Huang J. H. Huang Z. L. (2023b). Camrelizumab (a PD-1 inhibitor) plus apatinib (an VEGFR-2 inhibitor) and hepatic artery infusion chemotherapy for hepatocellular carcinoma in Barcelona Clinic Liver Cancer stage C (TRIPLET): a phase II study. Signal Transduct. Target Ther. 8, 413. 10.1038/s41392-023-01663-6 37884523 PMC10603153

[B37] Zhang W. Tong S. Hu B. Wan T. Tang H. Zhao F. (2023a). Lenvatinib plus anti-PD-1 antibodies as conversion therapy for patients with unresectable intermediate-advanced hepatocellular carcinoma: a single-arm, phase II trial. J. Immunother. Cancer 11, e007366. 10.1136/jitc-2023-007366 37730273 PMC10514649

[B38] Zhao M. Guo Z. Zou Y. H. Li X. Yan Z. P. Chen M. S. (2024). Arterial chemotherapy for hepatocellular carcinoma in China: consensus recommendations. Hepatol. Int. 18, 4–31. 10.1007/s12072-023-10599-6 37864725

[B39] Zheng K. Zhu X. Fu S. Cao G. Li W. Q. Xu L. (2022). Sorafenib plus hepatic arterial infusion chemotherapy versus sorafenib for hepatocellular carcinoma with major portal vein tumor thrombosis: a randomized trial. Radiology 303, 455–464. 10.1148/radiol.211545 35103539

[B40] Zhu X. D. Huang C. Shen Y. H. Ji Y. Ge N. L. Qu X. D. (2021). Downstaging and resection of initially unresectable hepatocellular carcinoma with tyrosine kinase inhibitor and anti-PD-1 antibody combinations. Liver Cancer 10, 320–329. 10.1159/000514313 34414120 PMC8339461

