# Supplementary material for: Efficacy and safety of HAIC combined with tyrosine kinase inhibitors versus HAIC monotherapy for advanced hepatocellular carcinoma: a multicenter propensity score matching analysis
Source: Front Pharmacol. 2024 Jul 31;15:1410767. doi: 10.3389/fphar.2024.1410767 (PMC11322119; doi:10.3389/fphar.2024.1410767)
Supplement: Supplementary file 1 [file DataSheet1.docx]

Supplementary Material

# Supplementary Figures and Tables

## Supplementary Figure 1


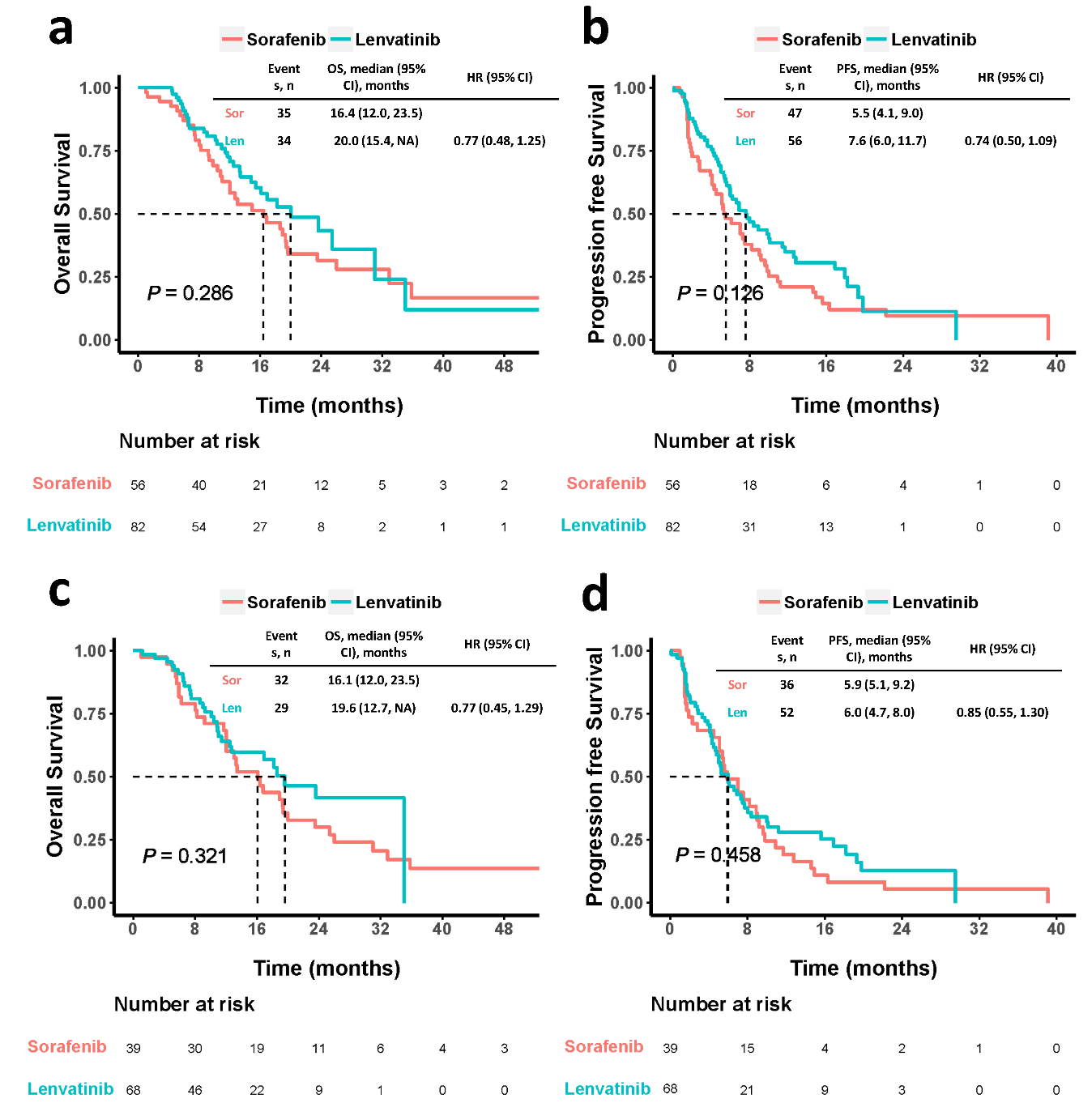


**Supplementary Figure 1.** Kaplan-Meier curves comparing OS and PFS among patients who underwent HAIC combined with lenvatinib versus HAIC combined with sorafenib before (a-b) and after (c-d) PSM.

*P* values were calculated using Log-rank test. PSM, propensity score matching; HT, HAIC combined with TKIs; HAIC: hepatic arterial infusion chemotherapy; TKIs, tyrosine kinase inhibitors; OS, overall survival; PFS: progression-free survival; HR: hazard ratio; CI: confidence interval; NA: not available.

## Supplementary Figure 2


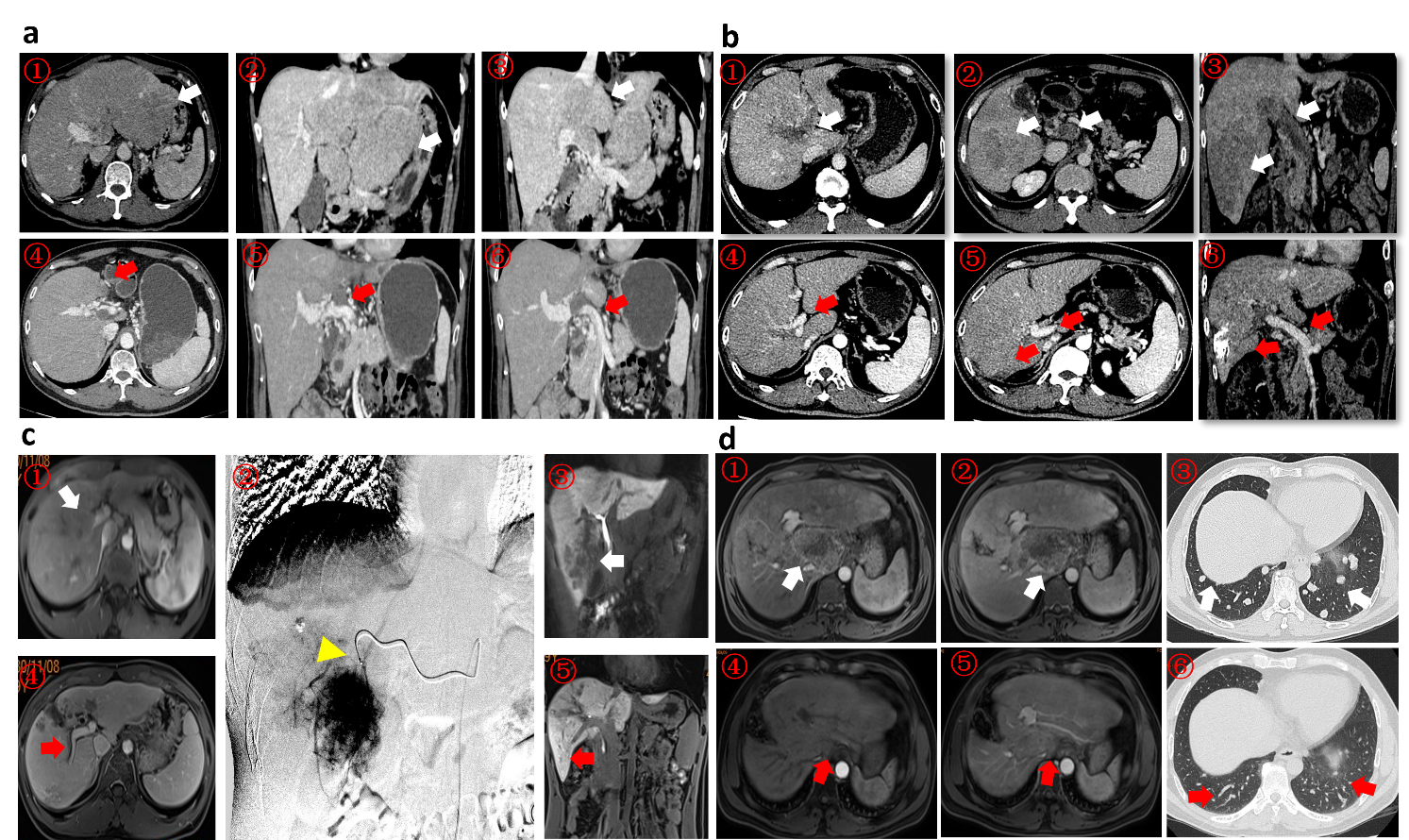


**Supplementary Figure 2.** The representative images of CR in the HA group (a-c) and in the HT group (d), respectively. a. Images in a 52-year-old man with hepatocellular carcinoma (HCC) with portal vein tumor thrombus (PVTT) and lymphatic metastasis who underwent HAIC monotherapy. ①&②&③ Contrast-enhanced CT images obtained at the hepatic arterial phase showed huge hypervascular masses in left lobe with PVTT and lymphatic metastasis (white arrow) before therapy; ④&⑤&⑥ Contrast-enhanced CT image obtained 12 weeks after 4 cycles of HAIC showed disappeared lesion in atrophied left lobe (red arrows). b. Images in a 58-year-old man with HCC with PVTT who underwent HAIC monotherapy. ①&②&③ Axial and coronal contrast-enhanced CT images obtained at the portal vein phase showed huge mass in left lobe and PVTT (white arrow) before therapy; ④&⑤&⑥ Axial and coronal contrast-enhanced CT images obtained 12 weeks after 4 cycles of HAIC showed disappeared lesion (red arrows). c. Images in a 61-year-old man with HCC and PVTT who underwent HAIC monotherapy. ①&③ Axial and coronal contrast-enhanced MR images obtained at the portal vein phase showed hypovascular masses with PVTT (white arrow) in right lobe before therapy; ② Digital subtraction angiography (DSA) image of HAIC, and the location of the microcatheter during HAIC (yellow triangle); ④&⑤ Axial and coronal contrast-enhanced MR images obtained 6 weeks after 2cycles of HAIC showed disappeared lesions and PVTT (red arrows). d. Images in a 55-year-old man with HCC and lung metastasis who underwent HAIC combined with lenvatinib. ①&② Axial contrast-enhanced MR images obtained at the hepatic arterial phase and portal vein phase showed huge hypervascular masses (white arrow) in caudate lobe before therapy; ③ Axial CT scan image showed multiple metastasis (white arrow) ④&⑤ Axial contrast-enhanced MR image obtained at the hepatic arterial phase and portal vein phase 12 weeks after 4 cycles of HAIC showed disappeared lesion (red arrows). ⑥ Axial CT scan image showed multiple metastasis disappeared (red arrow).

## Supplementary Figure 3


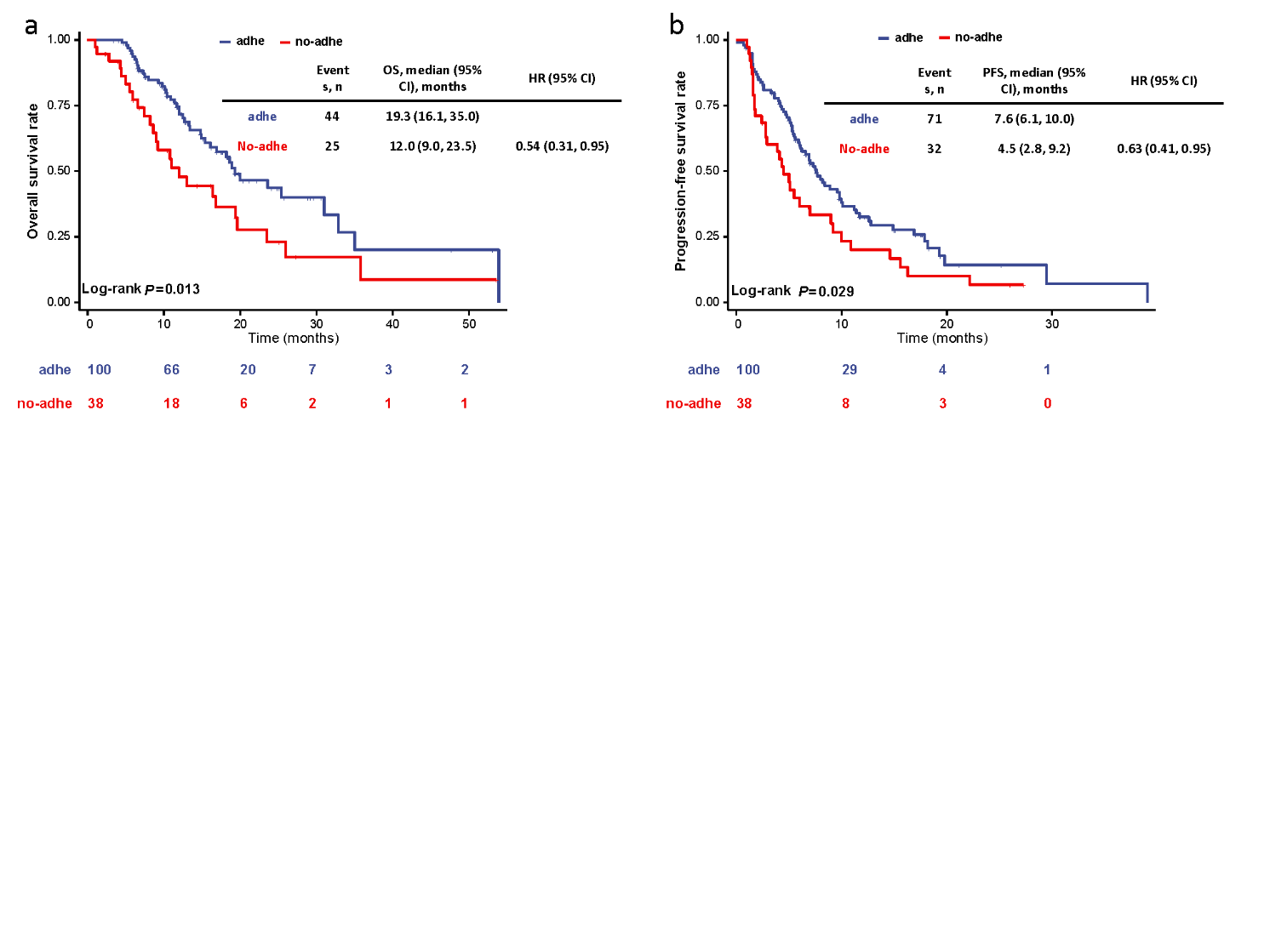


**Supplementary Figure 3.** Kaplan-Meier curves comparing OS (a) and PFS (b) between adherence to the TKI and reduction or discontinuation of the TKI in the HT group

*P* values were calculated using Log-rank test. PSM, propensity score matching; HT, HAIC combined with TKIs; HAIC: hepatic arterial infusion chemotherapy; TKIs, tyrosine kinase inhibitors; OS, overall survival; PFS: progression-free survival; HR: hazard ratio; CI: confidence interval; adhe referred to patients’ adherence to the TKI, and no-adhe referred to patients’ reduction or discontinuation of the TKI in the HT group.

## Supplementary Table 1

**Supplementary table 1** Baseline characteristics of the study patients before and after PSM

| **Characteristics** | **Before matching** | | | |  | **After matching** | | | |
| --- | --- | --- | --- | --- | --- | --- | --- | --- | --- |
|  | **AYYH**  **(n = 127)** | **SYMH**  **(n = 118)** | **TAH**  **(n = 91)** | ***P*** |  | **AYYH**  **(n =82)** | **SYMH**  **(n = 73)** | **TAH**  **(n = 59)** | ***P*** |
| Treatment |  |  |  | 0.26 |  |  |  |  | 0.23 |
| HA | 82 | 65 | 51 |  |  | 45 | 38 | 24 |  |
| HT | 45 | 53 | 40 |  |  | 37 | 35 | 35 |  |
| Age (mean ± SD, year) | 51 ± 12 | 50 ± 9 | 52 ± 10 | 0.82 |  | 50 ± 11 | 49 ± 10 | 51 ± 8 | 0.78 |
| < 60y | 100 (79%) | 91 (77%) | 65 (71%) | 0.44 |  | 63 (77%) | 60 (82%) | 44 (74%) | 0.54 |
| ≥ 60y | 27 (21%) | 27 (23%) | 26 (29%) |  |  | 19 (23%) | 13 (18%) | 15 (26%) |  |
| Sex |  |  |  | 0.66 |  |  |  |  | 0.71 |
| Male | 113 (89%) | 108 (92%) | 84 (92%) |  |  | 75 (91%) | 67 (92%) | 56 (95%) |  |
| Female | 14 (11%) | 10 (8%) | 7 (8%) |  |  | 7 (9%) | 6 (8%) | 3 (5%) |  |
| ECOG-PS |  |  |  | 0.49 |  |  |  |  | 0.86 |
| 0 | 108 (85%) | 103 (87%) | 74 (81%) |  |  | 72 (88%) | 66 (90%) | 52 (88%) |  |
| 1 | 19 (15%) | 15 (13%) | 17 (19%) |  |  | 10 (12%) | 7 (10%) | 7 (12%) |  |
| Etiology |  |  |  | 0.77 |  |  |  |  | 0.83 |
| HBV | 118 (93%) | 110 (93%) | 86 (94%) |  |  | 76 (93%) | 69 (94%) | 56 (95%) |  |
| HCV | 1 (1%) | 0 (0) | 0 (0) |  |  | 0 (0) | 0 (0) | 0 (0) |  |
| No-hepatitis | 8 (6%) | 8 (7%) | 5 (6%) |  |  | 6 (7%) | 4 (6%) | 3 (5%) |  |
| ALBI |  |  |  | 0.69 |  |  |  |  | 0.84 |
| 1 | 50 (39%) | 38 (32%) | 35 (38%) |  |  | 33 (40%) | 27 (37%) | 21 (36%) |  |
| 2 | 76 (60%) | 79 (67%) | 56 (62%) |  |  | 49 (60%) | 46 (63%) | 38 (64%) |  |
| 3 | 1 (1%) | 1 (1%) | 0 (0) |  |  | 0 (0) | 0 (0) | 0 (0) |  |
| Child-Pugh |  |  |  | 0.89 |  |  |  |  | 0.86 |
| A | 114 (90%) | 108 (92%) | 82 (90%) |  |  | 76 (93%) | 68 (93%) | 56 (95%) |  |
| B | 13 (10%) | 10 (8%) | 9 (10%) |  |  | 6 (7%) | 5 (7%) | 3 (5%) |  |
| Size (Mean ± SD, cm) | 12.8 ± 8.6 | 11.8 ± 9.6 | 11.5 ± 6.8 | 0.61 |  | 12.4 ± 7.8 | 11.9 ± 8.8 | 11.8 ± 9.6 | 0.73 |
| < 10cm | 33 (26%) | 35 (30%) | 31 (34%) | 0.74 |  | 18 (22%) | 20 (27%) | 17 (29%) | 0.81 |
| 10–15cm | 70 (55%) | 64 (54%) | 44 (48%) |  |  | 48 (59%) | 42 (58%) | 34 (58%) |  |
| ≥ 15cm | 24 (19%) | 19 (16%) | 16 (18%) |  |  | 16 (19%) | 11 (15%) | 8 (13%) |  |
| Number |  |  |  | 0.76 |  |  |  |  | 0.99 |
| Single | 41 (32%) | 33 (28%) | 28 (31%) |  |  | 30 (37%) | 26 (36%) | 21 (36%) |  |
| Multiple | 86 (68%) | 85 (72%) | 63 (69%) |  |  | 52 (63%) | 47 (64%) | 38 (64%) |  |
| AFP (μg/L) |  |  |  | 0.77 |  |  |  |  | 0.80 |
| ≤ 400 | 42 (33%) | 36 (30%) | 32 (35%) |  |  | 27 (33%) | 22 (30%) | 21 (36%) |  |
| > 400 | 85 (67%) | 82 (70%) | 59 (65%) |  |  | 55 (67%) | 51 (70%) | 38 (64%) |  |
| PVTT |  |  |  | 0.66 |  |  |  |  | 0.42 |
| Presence | 97 (76%) | 94 (80%) | 74 (81%) |  |  | 66 (80%) | 64 (88%) | 51 (86%) |  |
| Absence | 30 (24%) | 24 (20%) | 17 (19%) |  |  | 16 (20%) | 9 (12%) | 8 (14%) |  |
| Extrahepatic metastasis |  |  |  | 0.56 |  |  |  |  | 0.81 |
| Presence | 72 (57%) | 73 (62%) | 50 (55%) |  |  | 43 (52%) | 42 (57%) | 32 (54%) |  |
| Absence | 55 (43%) | 45 (38%) | 41 (45%) |  |  | 39 (48%) | 31 (43%) | 27 (46%) |  |

Values are presented as n (%).

*P* values were calculated using a two-sided χ^2^ test.

PSM, propensity score matching; HA, HAIC monotherapy; HAIC, hepatic arterial infusion chemotherapy; HT, HAIC combined with TKIs; TKIs, tyrosine kinase inhibitors; ECOG, Eastern Cooperative Oncology Group; PS, performance score; HBV, hepatitis B virus; HCV, hepatitis C virus; ALBI, albumin-bilirubin; AFP, alpha-fetoprotein; PVTT, portal vein tumor thrombus; AYYH, The Affiliated Yantai Yuhuangding Hospital of Qingdao University; SYMH, Sun Yat-sen Memorial Hospital, Sun Yat-sen University; TAH, The Third Affiliated Hospital of Sun Yat-sen University.

## Supplementary Table 2

**Supplementary table 2** The category of TKIs and number of patients in the HT group

| **TKIs category** |  | **Before matching**  **(n = 138)** | **After matching**  **(n = 107)** |
| --- | --- | --- | --- |
| **Sorafenib** |  | 56 (40.6%) | 39 (36.4%) |
| **Lenvatinib** |  | 82 (59.4%) | 68 (63.6%) |

Values are presented as n (%).

HT, HAIC combined with TKIs; TKIs, tyrosine kinase inhibitors.
